# Analysis of autonomic outcomes in APOLLO, a phase III trial of the RNAi therapeutic patisiran in patients with hereditary transthyretin-mediated amyloidosis

**DOI:** 10.1007/s00415-019-09602-8

**Published:** 2019-11-14

**Authors:** Alejandra González-Duarte, John L. Berk, Dianna Quan, Michelle L. Mauermann, Hartmut H. Schmidt, Michael Polydefkis, Márcia Waddington-Cruz, Mitsuharu Ueda, Isabel M. Conceição, Arnt V. Kristen, Teresa Coelho, Cécile A. Cauquil, Céline Tard, Madeline Merkel, Emre Aldinc, Jihong Chen, Marianne T. Sweetser, Jing Jing Wang, David Adams

**Affiliations:** 1 Instituto Nacional de Ciencias Médicas Y Nutrición Salvador Zubirán, Vasco de Quiroga 15, Sección XVI, Tlalpan, CdMx, CP 01400 México City, Mexico; 2 Boston Medical Center, Boston, MA USA; 3 University of Colorado, Denver, CO USA; 4 Mayo Clinic, Rochester, MN USA; 5 University of Münster, Münster, Germany; 6 Johns Hopkins University School of Medicine, Baltimore, MD USA; 7 Hospital Universitário Clementino Fraga Filho-UFRJ, Rio de Janeiro, Brazil; 8 Kumamoto University Hospital, Kumamoto, Japan; 9 CHULN, Hospital de Santa Maria and Faculdade de Medicina, Universidade de Lisboa, Lisbon, Portugal; 10 University of Heidelberg, Heidelberg, Germany; 11 Hospital de Santo António, Centro Hospitalar Universitário Do Porto, Porto, Portugal; 12 AP-HP Université Paris Saclay, CHU Bicêtre, Le Kremlin Bicêtre, France; 13 Université de Lille, Lille, France; 14 Alnylam Pharmaceuticals, Cambridge, MA USA; 15 AP-HP, Université Paris Saclay, CHU Bicêtre, Université Paris-Sud, INSERM 1195, Paris, France

**Keywords:** Patisiran, Small interfering ribonucleic acid (siRNA), Hereditary transthyretin-mediated amyloidosis, Transthyretin, Autonomic nervous system diseases, Polyneuropathy

## Abstract

Hereditary transthyretin-mediated (hATTR) amyloidosis is a progressive, debilitating disease often resulting in early-onset, life-impacting autonomic dysfunction. The effect of the RNAi therapeutic, patisiran, on autonomic neuropathy manifestations in patients with hATTR amyloidosis with polyneuropathy in the phase III APOLLO study is reported. Patients received patisiran 0.3 mg/kg intravenously (*n* = 148) or placebo (*n* = 77) once every 3 weeks for 18 months. Patisiran halted or reversed polyneuropathy and improved quality of life from baseline in the majority of patients. At baseline, patients in APOLLO had notable autonomic impairment, as demonstrated by the Composite Autonomic Symptom Score-31 (COMPASS-31) questionnaire and Norfolk Quality of Life-Diabetic Neuropathy (Norfolk QOL-DN) questionnaire autonomic neuropathy domain. At 18 months, patisiran improved autonomic neuropathy symptoms compared with placebo [COMPASS-31, least squares (LS) mean difference, − 7.5; 95% CI: − 11.9, − 3.2; Norfolk QOL-DN autonomic neuropathy domain, LS mean difference, − 1.1; − 1.8, − 0.5], nutritional status (modified body mass index, LS mean difference, 115.7; − 82.4, 149.0), and vasomotor function (postural blood pressure, LS mean difference, − 0.3; − 0.5, − 0.1). Patisiran treatment also led to improvement from baseline at 18 months for COMPASS-31 (LS mean change from baseline, − 5.3; 95% CI: − 7.9, − 2.7) and individual domains, orthostatic intolerance (− 4.6; − 6.3, − 2.9) and gastrointestinal symptoms (− 0.8; − 1.5, − 0.2). Rapid worsening of all study measures was observed with placebo, while patisiran treatment resulted in stable or improved scores compared with baseline. Patisiran demonstrates benefit across a range of burdensome autonomic neuropathy manifestations that deteriorate rapidly without early and continued treatment.

**Electronic supplementary material:**

The online version of this article (10.1007/s00415-019-09602-8) contains supplementary material, which is available to authorized users.

## Introduction

Hereditary transthyretin-mediated (hATTR) amyloidosis, also known as ATTRv amyloidosis, is a rare, inherited, rapidly progressive, fatal disease [1–5] caused by mutations in the transthyretin (*TTR*) gene [2]. Pathogenic *TTR* mutations lead to the oligomer formation that accumulates as amyloid deposits in multiple tissues and organs, including nerves, heart, and gastrointestinal (GI) tract [4]. The patchy deposition of amyloid leads to heterogeneous neurologic (e.g., autonomic neuropathy) and cardiac manifestations [1, 6], with the majority of patients developing a mixed phenotype of polyneuropathy and cardiomyopathy [7–9]. Untreated hATTR amyloidosis rapidly progresses, leading to deteriorating quality of life (QOL) and loss of function [1–3, 10]. The overall median survival is 4.7 years following diagnosis [11], which is further reduced to 3.4 years for patients with cardiac manifestations [12].

Impairment of the autonomic nervous system is common in patients with hATTR amyloidosis and can manifest as disturbance of GI motility, sexual dysfunction, bladder dysfunction, cardiovascular (CV) symptoms, pupillomotor impairment, and/or vasomotor impairment [13–16]. Dysfunction of the peripheral autonomic nerves occurs more frequently in hATTR amyloidosis than in other types of amyloidoses (e.g., immunoglobulin light chain [AL] amyloidosis or amyloid A [AA] amyloidosis) [8, 13, 14, 17], with autonomic neuropathy reported in approximately half of patients with hATTR amyloidosis in the Transthyretin Amyloidosis Outcomes Survey (THAOS) registry. Autonomic dysfunction occurs with a variety of *TTR* mutations including those not historically associated with neuropathic manifestations (e.g., V122I) [8, 18]. Typically, autonomic dysfunction occurs in the early stages of disease, prior to gross motor impairment, and is especially predominant in patients with the V30M *TTR* mutation and early-onset disease [6, 8]. This autonomic impairment is due to injury of unmyelinated and small myelinated nerve fibers by amyloid deposits and circulating *TTR* oligomers. As the disease progresses [19], patients report a substantial effect on their QOL, limiting their participation in physical and social activities [20–23].

CV autonomic dysfunction can present as orthostatic hypotension/intolerance, dizziness, syncope, fatigue, and blurry vision while standing [20, 21, 24, 25], which significantly impacts patients’ daily routine [20, 21]. Autonomic denervation of the heart in hATTR amyloidosis [26, 27] affects sympathetic and/or parasympathetic regulation, which may lead to conduction disturbances and arrhythmias [13, 28–30]. As these CV manifestations may also be a result of amyloid infiltration and deposition in cardiac tissue [31], the autonomic nature of these manifestations may go unrecognized.

GI dysfunction is common in patients with hATTR amyloidosis [8, 23] resulting in symptoms including gastroparesis (e.g., early satiety, nausea, postprandial vomiting), paralytic ileus, and other gastrointestinal motility disorders (e.g., severe diarrhea/constipation) [32–34] that ultimately lead to progressive weight loss, dehydration, and severe malnutrition [16, 24, 33, 35, 36]. The resulting malabsorption and cachexia are known to be a leading cause of death in hATTR amyloidosis [37, 38]. Frequent diarrhea and fecal incontinence can have a significant negative impact on QOL [23, 38–40], leading to withdrawal from social situations [20, 41]. Additionally, sexual dysfunction, which patients have described as “devastating”, may also have a major impact on patients’ QOL and functioning [20, 21].

Despite its prevalence among patients with hATTR amyloidosis and its significant impact on patient QOL and survival, standardized assessment of autonomic dysfunction in these patients does not exist [16]. Appropriately assessing the multiple manifestations of hATTR amyloidosis, including autonomic dysfunction, was a key consideration for the design of the phase III APOLLO study [9]. In the APOLLO study, patisiran treatment led to significant improvements in multiple QOL and polyneuropathy endpoints versus placebo, while demonstrating an acceptable benefit:risk profile [9]. The objective of this analysis is to contextualize the burden of autonomic dysfunction in patients with hATTR amyloidosis and to report the impact of patisiran on this aspect of the disease from the APOLLO study results.

## Methods

The full methodology, including study design and statistical analyses for APOLLO, has been described previously [9, 42]; relevant details are summarized briefly below. All analyses presented here are based on the modified intention-to-treat (mITT) population, which includes all patients who were randomized and received at least one dose of study drug. Details on question-level analyses of several of the endpoints are provided in their respective sections. All measures of autonomic dysfunction described here were included as secondary or exploratory endpoints in the APOLLO phase III study.

### Study design and patients

APOLLO (NCT01960348) was a multicenter, international, randomized, double-blind, placebo-controlled, phase III study.

Eligible patients were aged 18–85 years with a documented *TTR* mutation and diagnosis of hATTR amyloidosis, polyneuropathy (Neuropathy Impairment Score [NIS]: 5–130), adequate liver and kidney function, and a polyneuropathy disability (PND) score ≤ IIIb. Patients were enrolled at 44 sites across 19 countries between December 2013 and January 2016, and randomized (2:1) to receive an intravenous (IV) infusion of 0.3 mg/kg patisiran or placebo, once every 3 weeks for 18 months. All eligible patients provided their informed consent prior to their enrollment in the study.

### Study assessments

#### Efficacy and safety

Details of the efficacy and safety results of patisiran in the APOLLO study have been described previously [9, 43], including the effect of patisiran on sensorimotor secondary endpoints (i.e., NIS-weakness [NIS-W] subdomain, Rasch-built Overall Disability Scale [R-ODS], and 10-meter walk test) and exploratory endpoints (e.g., nerve conduction studies five attributes subcomponent of mNIS + 7 [∑5 NCS], PND score, and FAP stage).

#### Measures of autonomic symptoms: COMPASS-31 and Norfolk QOL-DN autonomic neuropathy domain

Composite Autonomic Symptom Score-31 (COMPASS-31) questionnaire is a 31-question patient-reported outcome assessment that measures autonomic symptoms across six weighted domains (orthostatic intolerance [40 points]; vasomotor [5 points]; secretomotor [15 points]; gastrointestinal [25 points]; bladder [10 points]; and pupillomotor [5 points]), on a 100-point scale [44]; a higher score indicates worse autonomic dysfunction.

Within the GI domain and orthostatic intolerance domain of the COMPASS-31, exploratory question-level analyses were conducted to evaluate the impact of patisiran or placebo on diarrhea and orthostatic intolerance symptoms at 18 months, compared with each patient’s own baseline (categorized as improved, no change, worsened, or missing). Specific questions analyzed in the GI domain included: “In the past year, have you had any bouts of diarrhea?” (yes or no). If yes, “How severe are these bouts of diarrhea?” (mild, moderate, severe). Within the orthostatic intolerance domain, questions evaluated included, “In the past year, have you ever felt faint, dizzy, ‘goofy’, or had difficulty thinking soon after standing up from a sitting or lying position?” (yes or no). If yes, “How would you rate the severity of these feelings or symptoms?” (mild, moderate, severe).

The 35-item Norfolk Quality of Life-Diabetic Neuropathy (Norfolk QOL-DN) questionnaire was designed to assess QOL in patients with diabetic neuropathy. However, it has also been used to understand disease severity and impact on QOL in patients with hATTR amyloidosis [19, 42]. The autonomic neuropathy domain of this questionnaire (0–12 points) includes specific questions on vomiting, diarrhea, and dizziness and/or fainting, in which a higher score indicates worsening [19, 42].

Within the autonomic neuropathy domain of the Norfolk QOL-DN, an exploratory question-level analysis was conducted to evaluate the impact of patisiran and placebo on diarrhea symptom severity at 18 months compared with baseline. The specific question analyzed in the autonomic neuropathy domain included: “In the past 4 weeks, have you had a problem with diarrhea and/or loss of bowel control?” (not a problem, very mild problem, mild problem, moderate problem, severe problem).

#### Measure of nutritional status affected by gastrointestinal autonomic symptoms: mBMI

Modified body mass index (mBMI) is a measure of nutritional status and wasting due in part to GI dysfunction, which can result from autonomic nervous system impairment [38]. mBMI was calculated by serum albumin concentration (g/L) multiplied by conventional body mass index (BMI) (weight in kg divided by the square of height in m^2^). mBMI was chosen as patients with hATTR amyloidosis can have low serum albumin levels, due to malnutrition, which leads to fluid retention and edema resulting in increased weight and normal BMI measurements despite worsening nutritional status. A lower mBMI score indicates worse nutritional status (i.e., greater autonomic functional impairment) [9].

### Measure of autonomic CV function: postural blood pressure (mNIS + 7 component)

The modified Neuropathy Impairment Score + 7 (mNIS + 7) scale is a 304-point composite measure comprising five weighted components that has been specifically designed for use in patients with hATTR amyloidosis with polyneuropathy [45]. Within this scale, autonomic function is assessed by postural blood pressure (BP) scoring which is based on the change in systolic BP upon standing: systolic BP decrease of < 20 mmHg = 0 points; decrease of 20 to < 30 mmHg = 1 point; and decrease of ≥ 30 mmHg = 2 points [42]; a higher score reflects a worse degree of orthostatic intolerance.

### Statistical analyses

Efficacy endpoints were assessed longitudinally and analyzed using a restricted maximum likelihood (REML)-based mixed-effects model repeated measures (MMRM) method. The outcome variable was change from baseline. Baseline value was included as a continuous covariate, and treatment group, visit (Month 9 or 18), treatment-by-visit interaction, age at symptom onset (< 50 versus ≥ 50 years), geographic region (North America, Western Europe, and the Rest of World), genotype (V30M versus non-V30M), prior stabilizer use (yes versus no), and baseline NIS (< 50 versus ≥ 50) were included as fixed effect terms. The primary comparison was the contrast (difference in least squares [LS] means) between the patisiran and placebo groups at 18 months. Analyses were conducted using the SAS PROC MIXED software package.

## Results

### Patient disposition and baseline clinical characteristics

In total, 225 patients were randomized to receive patisiran (*n* = 148; 138 [93.2%] completed the trial) or placebo (*n* = 77; 55 [71.4%] completed the trial). The two groups were generally balanced with respect to baseline characteristics and disease severity as detailed in Adams et al. [9] and in Solomon et al. [46]. Baseline autonomic measurements of the overall population, including COMPASS-31, mBMI, the postural BP component of mNIS + 7, and the autonomic neuropathy domain of Norfolk QOL-DN are detailed in Table 1. These measures are also well balanced between treatment groups and indicate the notable autonomic impairment present at baseline in patients enrolled in APOLLO. Across all study endpoints, there was a lower percentage of missing data in the patisiran group than the placebo group due to a lower rate of treatment discontinuation (7.4% versus 37.7%, respectively; reasons include: death, withdrawal, receipt of alternative therapy due to rapid disease progression, or random missingness).

**Table 1:** Disease characteristics of autonomic endpoints at baseline

Characteristic	Placebo (*n* = 77)	Patisiran (*n* = 148)	Total (*n* = 225)
COMPASS-31 total score (range 0–100), mean (± SD)	30.3 (16.4)	30.6 (17.6)	30.5 (17.1)
Orthostatic intolerance (range: 0–40), mean (± SD)	13.2 (11.2)	14.2 (10.8)	13.8 (10.9)
Vasomotor (range 0–5), mean (± SD)	1.0 (1.4)	0.9 (1.44)	1.0 (1.4)
Secretomotor (range 0–15), mean (± SD)	4.9 (3.7)	4.2 (3.7)	4.4 (3.7)
Gastrointestinal (range 0–25), mean (± SD)	8.1 (3.5)	8.2 (4.3)	8.1 (4.1)
Bladder (range 0–10), mean (± SD)	1.9 (2.7)	2.0 (2.5)	2.0 (2.5)
Pupillomotor (range 0–5), mean (± SD)	1.1 (1.1)	1.2 (1.2)	1.2 (1.2)
mBMI (kg/m^2^ × g/L), mean (± SD)	989.9 (214.2)	969.7 (210.5)	976.6 (211.5)
BMI (kg/m^2^), mean (± SD)	23.6 (4.3)	23.0 (4.4)	23.2 (4.4)
Albumin (g/dL), mean (± SD)	41.8 (3.4)	42.1 (3.5)	42.0 (3.5)
Weight (kg), mean (± SD)	67.5 (15.7)	67.3 (16.6)	67.4 (16.3)
mNIS + 7 total score (range 0–304), mean (± SD)	74.6 (37.0)	80.9 (41.5)	78.8 (40.1)
Postural blood pressure (range 0–2), mean (± SD)	0.6 (0.7)	0.7 (0.8)	0.6 (0.8)
Norfolk QOL-DN total score (range − 4 to 136), mean (± SD)	55.5 (24.3)	59.6 (28.2)	58.3 (27.0)
Autonomic neuropathy domain (range 0–12), mean (± SD)	2.9 (2.9)	3.0 (2.8)	3.0 (2.8)

*BMI* body mass index, *COMPASS-31* Composite Autonomic Symptom Score-31, *mBMI* modified body mass index, *mNIS* + *7* modified Neuropathy Impairment Score + 7, *Norfolk QOL-DN* Norfolk Quality of Life-Diabetic Neuropathy

### Measures of autonomic symptoms

#### COMPASS-31

**Fig. 1:**
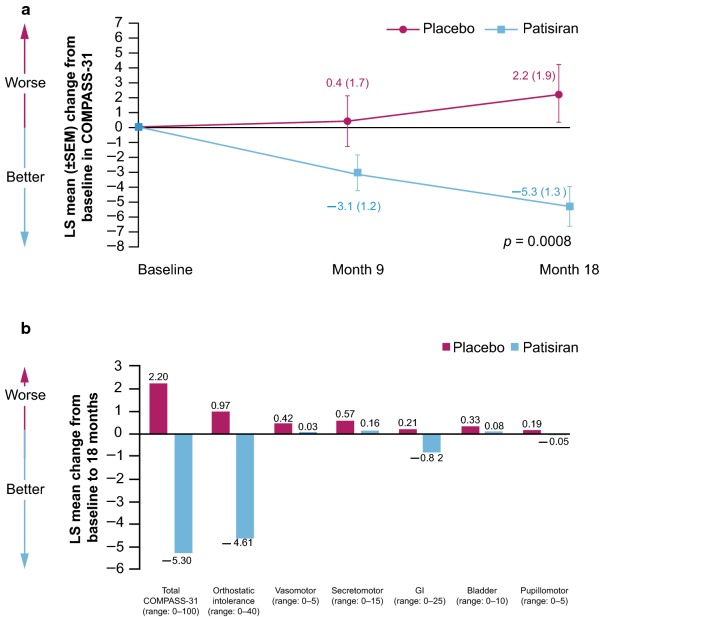
COMPASS-31 assessments in the APOLLO study. **a** LS mean change in COMPASS-31 score from baseline to 18 months in patients receiving patisiran or placebo in the APOLLO study. **b** LS mean change in individual COMPASS-31 domains from baseline to 18 months in the patisiran and placebo groups. *COMPASS-31* Composite Autonomic Symptom Score-31, *GI* gastrointestinal, *LS* least squares, *SEM* standard error of the mean

The mean (SD) total COMPASS-31 score at baseline was 30.6 (17.6) and 30.3 (16.4) points in the patisiran and placebo groups, respectively (Table 1). Total COMPASS-31 score improved in patisiran-treated patients compared with placebo-treated patients at 9 months, and reached statistical significance at 18 months, with an LS mean difference of − 7.5 (95% CI: − 11.9, − 3.2; *p* = 0.0008; Fig. 1a). Total COMPASS-31 score improved from baseline in patients who received patisiran at 9 and 18 months (LS mean change of − 3.1 points [95% CI: − 5.5, − 0.7] and − 5.3 points [95% CI: − 7.9, − 2.7], respectively) and worsened by 18 months in patients who received placebo (2.2 point worsening [95% CI: − 1.6, 6.1]).

An improvement was also observed across each of the six autonomic domains of COMPASS-31. Overall, mean values for each COMPASS-31 component were either similar to or improved from baseline in the patisiran arm, but were similar to or worsened from baseline in the placebo arm. The orthostatic intolerance and GI (symptoms of gastroparesis, constipation, diarrhea) domains demonstrated improvement in patients receiving patisiran not only when compared with placebo treatment at 18 months, but also relative to their own baseline (LS mean change from baseline of − 4.6 [95% CI: − 6.3, − 2.9] for the orthostatic intolerance domain and − 0.8 [95% CI: − 1.5, − 0.2] for the GI symptoms domain) (Fig. 1b).

**Fig. 2:**
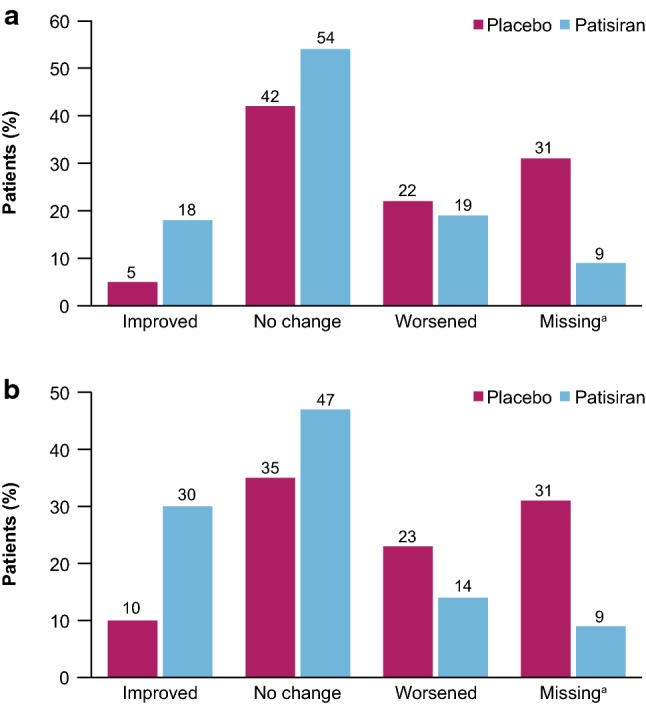
Question-level analysis of domains in COMPASS-31. **a** GI domain: change from baseline in diarrhea presence and severity at Month 18. **b** Orthostatic intolerance domain: change from baseline in orthostatic intolerance presence and severity at Month 18. ^a^Missing data at 18 months were more common in the placebo group (*n* = 24, 31% overall) than the patisiran group (*n* = 13, 9% overall). Reasons for the missing data in this analysis include: placebo–death (*n* = 4), early withdrawal of subject (*n* = 15), incomplete data at baseline (*n* = 1), random missingness (*n* = 4); patisiran: death (*n* = 6), early withdrawal of subject (*n* = 4), incomplete data at baseline (*n* = 3). *COMPASS-31* Composite Autonomic Symptom Score-31, *GI* gastrointestinal

At baseline, severity of diarrhea and orthostatic intolerance symptoms were well balanced. After 18 months of treatment, question-level analysis revealed that the patisiran group was 3.5-fold more likely to report improvement in severity of diarrhea than the placebo group (18% versus 5%, respectively; Fig. 2a) and threefold more likely than the placebo group to report improvement in severity of orthostatic intolerance (30% versus 10%, respectively; Fig. 2b). Additionally, the patisiran group was less likely to report worsening of orthostatic intolerance symptoms compared with the placebo-treated patients after 18 months (14% versus 23%, respectively; Fig. 2b).

### Norfolk QOL-DN: autonomic neuropathy domain

Norfolk QOL-DN total and autonomic neuropathy domain scores at baseline are shown in Table 1. At 18 months, a significant improvement was observed in total Norfolk QOL-DN score for patisiran-treated patients compared with the placebo group, expressed as a difference in LS mean change from baseline of − 21.1 points (95% CI: − 27.2, − 15.0; *p* = 1.10 × 10^–10^).

The Norfolk QOL-DN autonomic neuropathy domain assessment favored patisiran treatment, with an LS mean (standard error of the mean [SEM]) change from baseline at 18 months in this domain of − 0.6 (0.2) and + 0.5 (0.3) in the patisiran and placebo arms, respectively. LS mean difference of patisiran compared with placebo for this domain was − 1.1 (95% CI: − 1.8, − 0.5; *p* = 0.001). The consistent effects in favor of patisiran treatment compared with placebo were observed in all Norfolk QOL-DN domains in the overall population.

At 18 months, results of the question-level analysis showed that more placebo-treated patients reported moderate or severe diarrhea and/or loss of bowel control in the past 4 weeks compared with baseline (43% versus 33%, respectively). By comparison, fewer patisiran patients reported moderate or severe symptoms in the past 4 weeks at 18 months compared with baseline (27% versus 34%, respectively) (Supplementary Table 1).

### Measure of nutritional status affected by gastrointestinal autonomic symptoms: mBMI

**Fig. 3:**
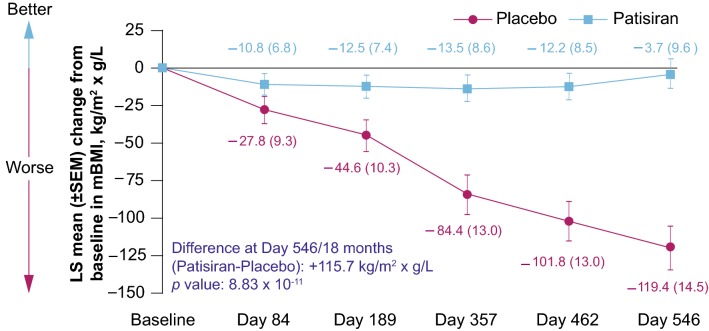
Change in least squares mean total mBMI score from baseline in patisiran and placebo groups over time (mITT population). The difference between placebo and patisiran at 18 months was + 115.7 kg/m^2^ × g/L (*p* = 8.83 × 10^–11^). *LS* least squares, *mBMI* modified body mass index, *mITT* modified intention-to-treat, *SEM* standard error of the mean

mBMI values at baseline are shown in Table 1. At 18 months, mBMI was significantly improved in the patisiran-treated patients compared with placebo-treated patients, with an LS mean difference of + 115.7 (95% CI: − 82.4, 149.0; *p* = 8.83 × 10^−11^; Fig. 3). The favorable effect of patisiran on mBMI relative to placebo was observed at the first assessment (3 months) with nominal significance achieved by Day 189 (Fig. 3). At 18 months, 41.2% of the patisiran group demonstrated improvement in mBMI (defined as > 0 kg/m^2^ × g/L increase from baseline) compared with 6.5% of the placebo group. Furthermore, improvement with patisiran compared with placebo treatment was also observed with BMI (Supplementary Table 2).

### Measures of autonomic CV function: postural BP (mNIS + 7)

mNIS + 7 total scores and postural BP at baseline are shown in Table 1. An improvement in each individual component of mNIS + 7, including postural BP (range of scores: 0–2 points), was achieved with patisiran compared with placebo treatment at 18 months. At 18 months, the LS mean difference in postural BP score change between treatment groups was − 0.3 points (95% CI: − 0.5, − 0.1) favoring patisiran and, within the patisiran and placebo arms, the LS mean (SEM) change from baseline was − 0.2 (0.1) and + 0.1 (0.1), respectively.

## Discussion

Autonomic dysfunction is a common, debilitating aspect of hATTR amyloidosis that often manifests early in the course of the disease and has substantial adverse impact on QOL and survival. Amyloid injures unmyelinated and small myelinated nerves giving rise to autonomic dysfunction, in addition to amyloid infiltration in the heart and GI tract. The clinical heterogeneity of this disease contributes to the mixed phenotype that develops in the majority of patients with hATTR amyloidosis and necessitates the use of multiple assessments to fully capture the impact of treatment on the autonomic neuropathy caused by the disease.

Results from the APOLLO study emphasize the impact of autonomic dysfunction on QOL, everyday activities, and physical functioning in patients with hATTR amyloidosis. At baseline, the enrolled population exhibited more autonomic deficits (worse scores for COMPASS-31 and Norfolk QOL-DN autonomic neuropathy domain) than those observed in healthy volunteers [47]. For example, a mean COMPASS-31 score of 8.9 has been reported in healthy volunteers, compared with scores of approximately 30 at baseline in the APOLLO study [47]. Importantly, hATTR amyloidosis adversely affected nearly all of the individual COMPASS-31 domains. Similarly, APOLLO patients experienced worse scores at baseline for the Norfolk QOL-DN autonomic neuropathy domain (2.9–3.0) compared with mean scores of 0.1 reported in healthy volunteers [19]. Moreover, APOLLO patients reported similar scores at baseline to patients with diabetes who have self-reported DN and have reported at least one episode of ulceration, gangrene, or amputation (score of 3.2) [48]. These comparisons highlight the significant impact of autonomic dysfunction on QOL in patients with hATTR amyloidosis relative to healthy individuals and those with neuropathies with a different etiology.

Orthostatic intolerance poses a considerable risk to patients with hATTR amyloidosis because the lightheadedness or syncope associated with the condition may lead to falls and injury in an already frail population. These events can occur during low-intensity activity (e.g., getting out of bed), thus impacting daily living. GI symptoms have also been shown to affect the QOL of patients with hATTR amyloidosis, and objective measures of GI manifestations (i.e., wasting via mBMI) and their associated symptoms (patient-reported symptoms via Norfolk-QOL-DN and COMPASS-31) all demonstrated rapid deterioration in the placebo arm of the APOLLO study.

In addition to the clear effects on QOL, autonomic dysfunction remains a major concern in hATTR amyloidosis due to its association with mortality. For example, malnutrition and GI events have been shown to be prognostic factors for worse survival in patients with hATTR amyloidosis and were a significant cause of death in a long-term study of patients with this disease [23, 37, 39]. CV autonomic dysfunction is recognized as a particularly dangerous manifestation of dysautonomia, with sudden death recorded in patients with hATTR amyloidosis with impaired sympathetic and parasympathetic responses [13]. A similar association between CV autonomic instability and increased CV mortality has been documented in diabetes [49].

In the phase III APOLLO study, patisiran treatment consistently improved autonomic symptoms, with change from baseline in scores for the COMPASS-31 questionnaire and the Norfolk QOL-DN autonomic neuropathy domain both better with patisiran compared with placebo at 18 months. Both measures were also improved with patisiran relative to baseline. Patisiran treatment demonstrated improvement in orthostatic intolerance and GI dysfunction domains of COMPASS-31 at 18 months, compared with placebo and baseline, which is notable based on the impact these events have on patient QOL. Patisiran-treated patients also showed evidence of improvement from baseline in postural BP score. This finding was supported by patient-reported improvement observed in orthostatic intolerance following patisiran treatment. Finally, stabilization of nutritional status, mBMI, relative to baseline over 18 months was also observed.

In contrast with the patisiran group, the placebo group demonstrated rapid and continued deterioration in autonomic disease over 18 months in the APOLLO study. This rapid disease progression aligns with natural history studies of this disease that have shown worsening polyneuropathy over time [3].

Measures of sudomotor and genitourinary dysfunction (neurogenic bladder and sexual dysfunction) were not included in measures of autonomic function analyzed in this study, although these are also major symptoms of autonomic dysfunction. Alternative measures may also have provided greater insight into autonomic CV function. While heart rate with deep breathing (HRdb) was included as part of the mNIS + 7 scale, these data were excluded due to missing data, as almost one-third of patients could not be assessed due to a history of electronic pacing or cardiac arrhythmia.

The APOLLO study demonstrated that, in patients with hATTR amyloidosis with polyneuropathy, patisiran improved autonomic dysfunction in addition to measures of sensorimotor neuropathy and QOL [9], supporting its benefit for these patients. The early-onset and rapid progression of dysautonomia in hATTR amyloidosis highlights the urgency of both early diagnosis and early effective treatment to limit or prevent the accumulation of disease burden.

## Electronic supplementary material

Below is the link to the electronic supplementary material.

Supplementary file1 (DOCX 34 kb)
