# Correction to: Analysis of autonomic outcomes in APOLLO, a phase III trial of the RNAi therapeutic patisiran in patients with hereditary transthyretin-mediated amyloidosis

**DOI:** 10.1007/s00415-020-09715-5

**Published:** 2020-02-07

**Authors:** Alejandra González-Duarte, John L. Berk, Dianna Quan, Michelle L. Mauermann, Hartmut H. Schmidt, Michael Polydefkis, Márcia Waddington-Cruz, Mitsuharu Ueda, Isabel M. Conceição, Arnt V. Kristen, Teresa Coelho, Cécile A. Cauquil, Céline Tard, Madeline Merkel, Emre Aldinc, Jihong Chen, Marianne T. Sweetser, Jing Jing Wang, David Adams

**Affiliations:** 1 Instituto Nacional de Ciencias Médicas Y Nutrición Salvador Zubirán, Vasco de Quiroga 15, Sección XVI, Tlalpan, CdMx, CP 01400 México City, Mexico; 2 Boston Medical Center, Boston, MA USA; 3 University of Colorado, Denver, CO USA; 4 Mayo Clinic, Rochester, MN USA; 5 University of Münster, Münster, Germany; 6 Johns Hopkins University School of Medicine, Baltimore, MD USA; 7 Hospital Universitário Clementino Fraga Filho-UFRJ, Rio de Janeiro, Brazil; 8 Kumamoto University Hospital, Kumamoto, Japan; 9 CHULN, Hospital de Santa Maria and Faculdade de Medicina, Universidade de Lisboa, Lisbon, Portugal; 10 University of Heidelberg, Heidelberg, Germany; 11 Hospital de Santo António, Centro Hospitalar Universitário Do Porto, Porto, Portugal; 12 AP-HP Université Paris Saclay, CHU Bicêtre, Le Kremlin Bicêtre, France; 13 Université de Lille, Lille, France; 14 Alnylam Pharmaceuticals, Cambridge, MA USA; 15 AP-HP, Université Paris Saclay, CHU Bicêtre, Université Paris-Sud, INSERM 1195, Paris, France

## Correction to: Journal of Neurology 10.1007/s00415-019-09602-8

The original version of this article unfortunately contained a mistake in Table 1.

**Table 1:** Disease characteristics of autonomic endpoints at baseline

Characteristic	Placebo (*n* = 77)	Patisiran (*n* = 148)	Total (*n* = 225)
COMPASS-31 total score (range 0–100), mean (± SD)	30.3 (16.4)	30.6 (17.6)	30.5 (17.1)
Orthostatic intolerance (range: 0–40), mean (± SD)	13.2 (11.2)	14.2 (10.8)	13.8 (10.9)
Vasomotor (range 0–5), mean (± SD)	1.0 (1.4)	0.9 (1.44)	1.0 (1.4)
Secretomotor (range 0–15), mean (± SD)	4.9 (3.7)	4.2 (3.7)	4.4 (3.7)
Gastrointestinal (range 0–25), mean (± SD)	8.1 (3.5)	8.2 (4.3)	8.1 (4.1)
Bladder (range 0–10), mean (± SD)	1.9 (2.7)	2.0 (2.5)	2.0 (2.5)
Pupillomotor (range 0–5), mean (± SD)	1.1 (1.1)	1.2 (1.2)	1.2 (1.2)
mBMI (kg/m^2^ × g/L), mean (± SD)	989.9 (214.2)	969.7 (210.5)	976.6 (211.5)
BMI (kg/m^2^), mean (± SD)	23.6 (4.3)	23.0 (4.4)	23.2 (4.4)
Albumin (g/L), mean (± SD)	41.8 (3.4)	42.1 (3.5)	42.0 (3.5)
Weight (kg), mean (± SD)	67.5 (15.7)	67.3 (16.6)	67.4 (16.3)
mNIS + 7 total score (range 0–304), mean (± SD)	74.6 (37.0)	80.9 (41.5)	78.8 (40.1)
Postural blood pressure (range 0–2), mean (± SD)	0.6 (0.7)	0.7 (0.8)	0.6 (0.8)
Norfolk QOL-DN total score (range − 4 to 136), mean (± SD)	55.5 (24.3)	59.6 (28.2)	58.3 (27.0)
Autonomic neuropathy domain (range 0–12), mean (± SD)	2.9 (2.9)	3.0 (2.8)	3.0 (2.8)

*BMI* body mass index, *COMPASS-31* Composite Autonomic Symptom Score-31, *mBMI* modified body mass index, *mNIS* + *7* modified Neuropathy Impairment Score + 7, *Norfolk QOL-DN* Norfolk Quality of Life-Diabetic Neuropathy

Units for albumin were given as g/dL. It should be g/L.

