# Supplementary material for: Analysis of autonomic outcomes in APOLLO, a phase III trial of the RNAi therapeutic patisiran in patients with hereditary transthyretin-mediated amyloidosis
Source: J Neurol. 2019 Nov 14;267(3):703–12. doi: 10.1007/s00415-019-09602-8 (PMC7035216; doi:10.1007/s00415-019-09602-8)
Supplement: Supplementary file 1 — Supplementary file1 (DOCX 34 kb) [file 415_2019_9602_MOESM1_ESM.docx]

**Supplementary material**

**Supplementary Table 1** Question-level analysis of diarrhea question within autonomic neuropathy domain of Norfolk QOL-DN

| Treatment group | Problem with diarrhea and/or loss of bowel control | Baseline | | Month 18 | | Missing data at month 18 |
| --- | --- | --- | --- | --- | --- | --- |
|  |  | *n* | % | *n* | % | *n* |
| Placebo | Total | 76^a^ | 100 | 49 | 100 | 28^b^ |
|  | Moderate or severe problem | 25 | 33 | 21 | 43 |  |
|  | Moderate problem | 10 | 13 | 12 | 25 |  |
|  | Severe problem | 15 | 20 | 9 | 18 |  |
| Patisiran | Total | 147^a^ | 100 | 136 | 100 | 12^b^ |
|  | Moderate or severe problem | 50 | 34 | 37 | 27 |  |
|  | Moderate problem | 26 | 18 | 21 | 15 |  |
|  | Severe problem | 24 | 16 | 16 | 12 |  |

^a^In both patisiran and placebo groups, 1 patient had missing data at baseline

^b^Missing data at 18 months were more common in the placebo group (*n =* 28, 36% overall) than the patisiran group (*n =* 12, 8% overall). Reasons for the missing data in this efficacy evaluable mITT analysis include:

Placebo: death (*n =* 4); early withdrawal of subject (*n =* 16); receipt of alternative therapy (*n =* 6); random missingness (*n =* 2)

Patisiran: death (*n =* 6); early withdrawal of subject (*n =* 4); receipt of alternative therapy (*n =* 1); random missingness (*n =* 1)

Alternative therapy (local standard of care treatment for the polyneuropathy of hATTR amyloidosis) allowed per protocol in patients who exhibited rapid disease progression (defined as a ≥ 24-point increase in mNIS+7 and FAP stage progression relative to baseline) at 9 months

*FAP* familial amyloid polyneuropathy*, hATTR* hereditary transthyretin-mediated, *mITT* modified intention-to-treat, *mNIS+7* modified Neuropathy Impairment Score +7, *Norfolk QOL-DN* Norfolk Quality of Life-Diabetic Neuropathy

**Supplementary Table 2** Change in nutritional status in patients with hATTR amyloidosis in APOLLO. Components of mBMI

| Parameter measured | Placebo (*n* = 77) | Patisiran (*n* = 148) |
| --- | --- | --- |
| Serum albumin (g/L, mean [± SD]) | | |
| Baseline | 41.8 (3.4) | 42.1 (3.5) |
| At 18 months | 38.8 (4.3) | 41.3 (4.2) |
| % change from baseline at 18 months | −8.3 (7.6) | −2.6 (8.7) |
| BMI (kg/m^2^, mean [± SD]) | | |
| Baseline | 23.6 (4.3) | 23.0 (4.5) |
| At 18 months^a^ | 23.0 (4.4) | 23.4 (4.6) |
| Change from baseline at 18 months (LS mean [SEM])^b^ | −1.0 (0.2) 95% CI: −1.4, −0.6 | +0.4 (0.1) 95% CI: 0.1, 0.7 |
| Weight (kg, mean [± SD]) | | |
| Baseline | 67.5 (15.7) | 67.3 (16.6) |
| At 18 months^a^ | 66.3 (15.1) | 68.8 (17.1) |
| Mean change from baseline at 18 months | −3.1 (4.9) | +1.2 (4.8) |

^a^Day 546 is treated as Month 18 as for mBMI

^b^Difference patisiran–placebo: 1.4 kg/m^2^ (95% CI: 0.9, 1.9)

*BMI* body mass index, *hATTR* hereditary transthyretin-mediated, *LS* least squares, *mBMI* modified body mass index, *SD* standard deviation, *SEM* standard error of the mean
